# Identifying long non-coding RNAs involved in heat stress response during wheat pollen development

**DOI:** 10.3389/fpls.2024.1344928

**Published:** 2024-02-06

**Authors:** Saeid Babaei, Prem L. Bhalla, Mohan B. Singh

**Affiliations:** Plant Molecular Biology and Biotechnology Laboratory, School of Agriculture, Food and Ecosystem Sciences, The University of Melbourne, Melbourne, VIC, Australia

**Keywords:** wheat, lncRNA, heat stress, pollen development, transcriptome, pollen sterility

## Abstract

**Introduction:**

Wheat is a staple food crop for over one-third of the global population. However, the stability of wheat productivity is threatened by heat waves associated with climate change. Heat stress at the reproductive stage can result in pollen sterility and failure of grain development.

**Methods:**

This study used transcriptome data analysis to explore the specific expression of long non-coding RNAs (lncRNAs) in response to heat stress during pollen development in four wheat cultivars.

**Results and discussion:**

We identified 11,054 lncRNA-producing loci, of which 5,482 lncRNAs showed differential expression in response to heat stress. Heat-responsive lncRNAs could target protein-coding genes in *cis* and *trans* and in lncRNA-miRNA-mRNA regulatory networks. Gene ontology analysis predicted that target protein-coding genes of lncRNAs regulate various biological processes such as hormonal responses, protein modification and folding, response to stress, and biosynthetic and metabolic processes. We also noted some paired lncRNA/protein-coding gene modules and some lncRNA-miRNA-mRNA regulatory modules shared in two or more wheat cultivars. These modules were related to regulating plant responses to heat stress, such as heat-shock proteins and transcription factors, and protein domains, such as MADS-box, Myc-type, and Alpha crystallin/Hsp20 domain.

**Conclusion:**

Our results provide the basic knowledge and molecular resources for future functional studies investigating wheat reproductive development under heat stress.

## 1 Introduction

Wheat is one of the most important staple crops in the world and plays a crucial role in global food security. Wheat is cultivated on more land area than any other crop globally. It is a primary source of dietary energy and protein for a significant portion of the global population, particularly in regions where it is a staple food. Given its central role in global food and nutrition security, maintaining stable wheat production is crucial to ensuring a steady food supply for a growing human population. However, environmental challenges such as high temperatures and heat waves associated with climate change impair proper plant growth and development, decreasing crop yield and quality (Lippmann et al., 2019; El-Sappah et al., 2022). With an estimated 6% decrease in global productivity for each degree Celsius temperature increase, wheat (*Triticum aestivum*), a key cereal crop and staple food growing worldwide, is not an exception to this disruptive phenomenon (Shewry, 2009; Asseng et al., 2015; Lal et al., 2021). Heat stress is a significant challenge for wheat production, especially during the reproductive stage, which is most vulnerable to environmental challenges. The reproductive phase of wheat starts with the emergence of flag leaf and finishes with grain maturity, and any stress during this stage can profoundly affect yield and quality (Farooq et al., 2011; Nawaz et al., 2015). The production of viable pollen is an essential component of the reproductive development of a plant as it determines crop fertility and productivity (Singh and Bhalla, 2007). According to transcriptomic investigations (Haerizadeh et al., 2009; Babaei et al., 2021; Golicz et al., 2021), distinct expressions for various protein- and nonprotein-coding genes drive developmental processes that result in mature pollen. However, environmental stresses can interrupt these developmental processes, reducing fertility and yield; (Browne et al., 2021; Lohani et al., 2021; Singh et al., 2021). At the molecular level, stress can disrupt various physiological and biochemical processes in developing pollen, leading to altered gene expression, protein synthesis, reactive oxygen species (ROS) metabolism, lipid metabolism, carbohydrate metabolism, and hormone signaling.

Genetic variability in the heat stress response of pollen development has been reported among wheat cultivars (Dong et al., 2017; Browne et al., 2021), and three days of heat stress (35°C) disrupted pollen development in two heat-sensitive cultivars, Cranbrook and Wyalkathem but not in two tolerant cultivars, Halberd and Young (Browne et al., 2021). At the molecular level, differential expression of protein-coding genes has been observed during pollen development following heat stress in the above two sensitive and two tolerant wheat cultivars (Browne et al., 2021). High temperature strongly up-regulated HEAT SHOCK TRANSCRIPTION FACTOR A9 (HSFA9), BCL-2-ASSOCIATED ATHANOGENE 6 (BAG6) and FK Binding Protein 65 (FKBP65) genes in anthers of all cultivars. Many genes were up-regulated differentially in heat-sensitive and tolerant cultivars. Additionally, by comparing the genes up-regulated in heat-tolerant and sensitive cultivars, Browne et al. (2021) have identified several genes, including MYELOBLASTOSIS VIRAL ONCOGENE HOMOLOG 30 (MYB30), BAX INHIBITOR-1 (BI-1), and MULTIPROTEIN BRIDGING FACTOR 1 (MBF1), that may contribute to heat tolerance in developing anthers.

Much is known about the protein-coding genes involved in the pollen heat stress response in wheat, but little is known about the contribution of lncRNAs underlying the vulnerability of pollen development to high-temperature exposure. LncRNAs are transcripts over 200 nucleotides that do not code for proteins but have diverse and significant regulatory functions within the cells based on the sequence composition and structure (Golicz et al., 2018a; Wierzbicki et al., 2021).

LncRNAs have been reported as regulatory molecules in various cellular processes such as chromatin organization, transcription, splicing, mRNA stability, translation, and protein modification (Herman et al., 2022). Based on their molecular function, lncRNAs can be categorized into signal, decoy, guide, and scaffold molecules (Wang et al., 2011). LncRNAs show lower inter-species sequence conservation, lower expression, and higher tissue-specificity expression compared to protein-coding RNAs (Golicz et al., 2018a). LncRNAs are classified as sense, antisense, intronic, or intergenic based on their genomic location and transcriptional direction (Babaei et al., 2022). In addition, lncRNAs can be identified as “*cis*-acting” when they control gene expression at or close to their transcriptional site or “*trans*-acting” when they move from their transcriptional sites to act in other locations in the nucleus or cytoplasm (Gil and Ulitsky, 2020).

In plants, lncRNAs have also been shown to function in different biological processes during growth and development (Zhao et al., 2022). For example, lncRNA ALTERNATIVE SPLICING COMPETITOR (ASCO), which is involved in root development in *Arabidopsis*, play a role as a decoy lncRNA to hijack nuclear speckle RNA-binding proteins (NSR) regulating the splicing of several mRNA targets (Bardou et al., 2014). In rice, lncRNA TWISTED LEAF regulates the expression of R2R3 MYB transcription factor to play its regulatory role in maintaining the leaf blades flattened (Liu et al., 2018). For instance, a DROUGHT-INDUCED lncRNA (DRIR) has been reported as the positive regulator of drought and salt stress in *Arabidopsis* involved in the regulation of many stress-responsive genes (Qin et al., 2017). LncRNA SVALKA has also been shown to tightly regulate the expression of CBF1 in *Arabidopsis* and promote the plant’s ability to overcome severe cold stresses (Kindgren et al., 2018).

LncRNAs, as regulatory molecules, play crucial roles during pollen development and progression;(Xiao-Yan et al., 2004; Ding et al., 2012; Babaei et al., 2022). For example, sufficient expression of lncRNA LONG-DAY–SPECIFIC MALE-FERTILITY–ASSOCIATED RNA or in short, LDMAR is essential for pollen development and fertility in rice; even a single nucleotide mutation alters the secondary structure and expression of LDMAR, which leads to photoperiod-sensitive male sterility (Ding et al., 2012). Previously, the differential and specifical expression of several lncRNAs during pollen developmental stages has been reported in plants such as *Brassica rapa* (Huang et al., 2018; Lohani et al., 2023), *Camellia oleifera* (Kong et al., 2022), and *Oryza sativa* (Wang et al., 2021). During pollen development, lncRNAs can also be differently expressed in response to environmental challenges, which points to the regulatory role of lncRNAs as stress-responsive molecules. For instance, 131 heat-stress response lncRNAs were discovered during *Arabidopsis* pollen development (Rutley et al., 2021), and a total of 3,053 drought-stress responsive lncRNAs were found in tomato anthers (Lamin-Samu et al., 2022). However, the knowledge of the expression profile of stress-responsive lncRNAs during pollen development in wheat is still lacking. To this end, we performed *in-silico* analysis to identify and characterize 5,482 heat-responsive lncRNAs during wheat pollen development. We predicted the potential function of lncRNAs as *cis* and *trans*-regulatory RNAs in various biological processes. We also explored lncRNA interactions with miRNAs and predicted lncRNA-miRNA-mRNA regulatory networks in response to heat stress during pollen development in wheat.

## 2 Materials and methods

### 2.1 Data

The RNA sequencing data with accession numbers PRJNA638225 and PRJNA433429 from a previously published study (Browne et al., 2021) were retrieved from the Sequence Read Archive (SRA) database at the National Center for Biotechnology Information (https://www.ncbi.nlm.nih.gov/sra/). The RNA-seq data represents the transcriptome of four wheat cultivars at two stages of pollen development: meiosis and tetrad (**Supplementary Table S1**). Wheat cultivars were Cranbrook and Wyalkatchem for stress-sensitive and Halberd and Young for stress-tolerant samples. All cultivars were grown under normal conditions (day/night: 22°C/15°C) as control samples and heat stress conditions (day/night: 35°C (for 12 hours)/15°C).

The wheat genomic reference sequence (*Triticum aestivum*, IWGSC, release-55) and its corresponding annotation were downloaded from the Plant Ensemble database (https://plants.ensembl.org/).

### 2.2 LncRNA identification and differential expression analysis

Reads were mapped to the wheat reference genome using alignment software HISAT2-v2.2.1 (Kim et al., 2015). Aligned reads were assembled into full-length transcripts and subsequently merged using StringTie-v2.2.1 (Pertea et al., 2015). Gffcompare-v0.11.2 (Pertea and Pertea, 2020) was used to annotate assembled transcript, and then transcripts that were tagged as class-code “i”, “o”, “u” and “x” were selected. The genomic sequence of selected transcripts was obtained using BEDTools-v2.30.0 (Quinlan and Hall, 2010) for subsequent analysis. Transcripts with sequence lengths shorter than 200 nucleotides were removed. The remaining transcripts were screened for protein-coding potential using CPC2-v1.0.1 (labelled as non-coding) (Kang et al., 2017), CNCI-v2.0 (indexed as non-coding) (Sun et al., 2013), and CPAT-v3.0.4 (coding probability < 0.365) (Wang et al., 2013). For CPAT, the cut-off coding probability was calculated using the provided CPAT R-code and ROCR-v1.0-11 (Sing et al., 2005). Transcripts selected as non-coding using CPC, CNCI, and CPAT were scanned against the Pfam database for homolog identification using HMMER-v3.3.2 (Eddy, 2011) to remove the remaining protein-coding RNAs.

Kallisto-v0.46.2 (Bray et al., 2016) was used to quantify the abundance of all protein-coding and non-protein-coding transcripts. We summarized the transcript abundance to the gene level and analyzed the differential expression using NOISeq-v2.42.0 (Tarazona et al., 2015). Trimmed Mean of M-values (TMM) was used as the normalization method, and Count Per Million smaller than one (CPM < 1) was used to filter out low-count genes. Given the typically lower expression levels of lncRNAs compared to messenger RNAs (mRNAs) (Grammatikakis and Lal, 2022; Li et al., 2023), a threshold of significance was established for lncRNA differential expression, requiring a minimum |log2 fold change| > 0.5. Additionally, lncRNAs with a false discovery rate exceeding 0.05 were excluded. These criteria align with methodologies employed in previous studies for the selection of differentially expressed lncRNAs (Li et al., 2020; Ping et al., 2021; Lohani et al., 2023).

Pandas-v1.5.2 (The Pandas Development Team, 2022) handled and manipulated large tabular and text files. Plots were made using R-v4.2.2 (R Core Team, 2021) with libraries gplots-v3.1.3 (Warnes et al., 2022), ggplot2-v3.4.0 (Wickham, 2016), and VennDiagram-v1.7.3 (Chen and Boutros, 2011).

### 2.3 Identification of *cis* and *trans* targets of lncRNAs

The co-expression network between differentially expressed lncRNAs and protein-coding genes was constructed for functional lncRNA prediction. We used BEDTools to locate 10 genes at either side of lncRNA’s loci as their *cis* target; all other genes were considered to be the *trans* target. For the co-expression network, first, the CPM values were obtained using edgeR-v3.40.1 (Robinson et al., 2010). Then SciPy-v1.9.3 (Virtanen et al., 2020) was used to calculate the Pearson correlation coefficients (r) and corresponding *P* values (*P*). In *cis*, paired lncRNAs and protein-coding genes with *P* < 0.05 (|r| > 0.81) were selected. For *trans*-acting lncRNAs, MNE-Python-v1.3 (Gramfort et al., 2013) was used for multiple comparison correction using the Benjamini-Hochberg (BH) method, and paired lncRNAs and protein-coding genes with *P* < 0.05 were selected as co-expressed. As another filtering step for *trans*-acting lncRNAs, LncTar (Li et al., 2015) was used to measure the normalized binding free energy (ndG) between co-expressed lncRNA and its paired protein-coding gene. A cutoff of ndG = -0.15 was used to filter the LncTar results (Li et al., 2015; Zhao et al., 2021; Zhang et al., 2022).

### 2.4 Prediction of lncRNA-miRNA-mRNA regulatory network

The analysis of the interaction between lncRNAs and miRNAs and then miRNAs and mRNAs were also used to predict the function of differentially expressed lncRNAs. For this mean, known sequence of wheat miRNAs were retrieved from the miRBase database (Kozomara and Griffiths-Jones, 2010) and TargetFinder-v1.7 (https://github.com/carringtonlab/TargetFinder) (Fahlgren and Carrington, 2010) with its default parameters was used to identify RNA-RNA interactions.

### 2.5 Functional prediction of lncRNAs

For functional prediction, Gene Ontology (GO) enrichment analysis was carried out using topGO-v2.50.0 (Alexa, 2022) for all the identified *cis* and *trans* targets of lncRNAs as well as the genes that were placed in the lncRNA-miRNA-mRNA network. Significant GO identifiers (ClassicFisher < 0.05) were summarized to their parent GO terms using rrvgo-v1.10 (Sayols, 2020).

### 2.6 Conservation analysis of lncRNAs

For conservation analysis, the sequence information of lncRNAs identified in other plant species was retrieved from PLncDB v2.0 (https://www.tobaccodb.org/plncdb/) (Jin et al., 2021). Then BLASTN (-word_size 11) was used to align lncRNAs detected in this study against lncRNAs identified in other plant species. For a lncRNA to be conserved, the ‘E value’ had to be less than 1e-5, and the aligned lncRNAs had to contain a minimum of 60% identical nucleotides covering at least 30% of the length of shorter lncRNA in the alignment. The phylogenetic tree was constructed using MEGA 11 (Tamura et al., 2021).

## 3 Results

### 3.1 Genome-wide identification and characteristics of heat-stress associated lncRNAs in wheat pollen

**Figure 1:**
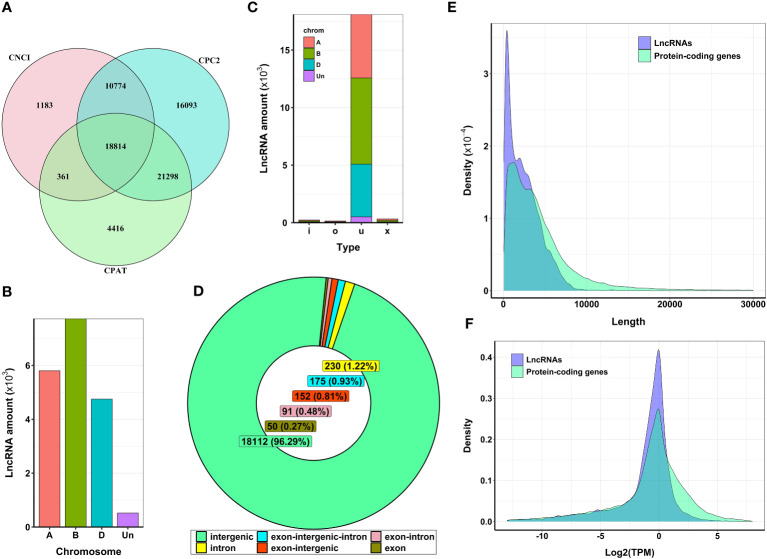
Features of expressed lncRNAs in wheat anthers. **(A)** Venn diagram representing the number of detected lncRNAs with no protein-coding potential. From 18,814 selected lncRNAs, four lncRNAs were filtered out using alignment against the Protein Families database (Pfam). **(B)** The distribution of lncRNAs generated from different sub-genomes. Most identified lncRNAs were generated from sub-genome **(B, C)** Type of lncRNAs identified in wheat. Most lncRNAs expressed from different wheat sub-genomes are classified as ‘u’, which means their genomic region is unknown or intergenic compared with known genomic regions. **(D)** The genomic location of lncRNA transcripts. The entire sequence of more than 96% of identified lncRNAs was derived from intergenic regions, and the remaining lncRNAs had some level of overlap with known protein-coding regions. **(E)** The length distribution of lncRNAs. The average length of lncRNAs was about 2,400 nucleotides. LncRNAs were shorter in length compared with protein-coding genes. **(F)** The expression level of lncRNAs and protein-coding genes. The mean log_2_ (TPM) values for lncRNAs were lower than those of protein-coding genes.

For the genome-wide discovery of heat stress-associated lncRNAs in wheat, we used publicly available RNA-seq libraries in the SRA database. The data represent the transcriptome of control and heat-stressed anthers at meiosis and the tetrad stages of pollen development in stress-tolerant (Halberd and Young) and stress-sensitive (Cranbrook and Wyalkatchem) wheat cultivars. The overall alignment rate of sequencing data to the wheat reference genome was more than 99% for all samples (**Supplementary Table S1**). Transcript assembly analysis and abundance quantification identified a total of 181,096 genomic loci, among which 42,536 loci were tagged as class-code “i”, “o”, “u”, and “x” and used for further lncRNA identification. Combining CPC2, CPAT, and CNCI methods (**Figure 1A**), our analysis detected 18,814 transcripts as potential lncRNAs. Further filtering against the Pfam database revealed four transcripts had protein-coding potential. Ultimately, we identified 18,810 transcripts (corresponding to 11,054 loci) as bona fide lncRNAs that lacked detectable protein-coding ability (**Supplementary Table S2**). 

Hexaploid wheat (2n=6x=42), which evolved from three ancestral genomes (A, B, and D), comprises 21 pairs of chromosomes. Our analysis of lncRNA transcription in this species revealed an uneven distribution of these transcripts across the A, B, and D genomes, as shown in **Figure 1B**. Most identified lncRNAs in our samples were in intergenic regions (96.29%), although a small fraction also overlapped with genic regions (**Figures 1C, D**). In terms of length, most lncRNAs ranged from 200 to 5,000 nucleotides, with an average length of approximately 2,400 nucleotides. Notably, this was shorter than the average length of protein-coding transcripts, about 4,200 nucleotides (**Figure 1E**). Finally, we observed that the average expression levels of lncRNAs were lower than those of protein-coding genes (**Figure 1F**).

### 3.2 Identification of 5,482 heat-responsive lncRNAs

**Figure 2:**
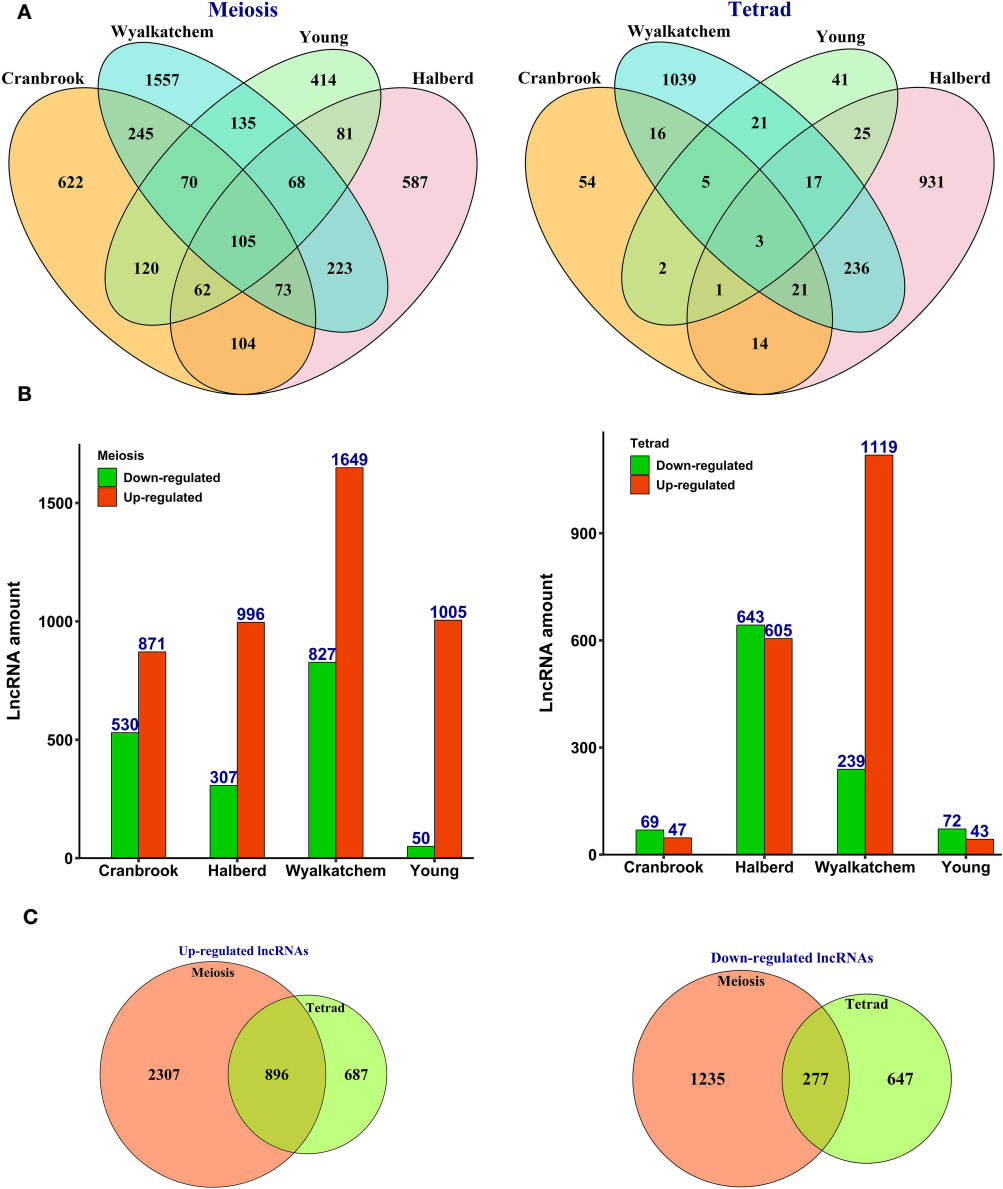
Expression pattern of heat-responsive lncRNAs during pollen development in heat-sensitive (Cranbrook and Wyalkatchem) or heat-tolerant (Halberd and Young) wheat cultivars. **(A)** The number of common and specific lncRNAs differentially expressed during meiosis or tetrad stages of pollen development. More lncRNAs showed cultivar-specific expression patterns, especially during the meiosis stage. **(B)** The number of lncRNAs with upregulation or downregulation trends in response to heat stress. LncRNAs had higher changes in their expression level with an upregulation trend in meiosis. **(C)** The number of stage-specific expressed lncRNAs. LncRNAs exhibited stage-specific expression patterns, with more lncRNAs showing differential expression in response to heat stress during meiosis than tetrad.

Further data analysis revealed that 5,482 lncRNAs were differentially expressed in response to heat stress in four wheat cultivars at meiosis and tetrad stages (**Supplementary Table S3**). We found that more lncRNAs showed differential expression patterns in meiosis than tetrad, with an up-regulation trend observed in all cultivars (**Figure 2A**). In tetrad, most of the differentially expressed lncRNAs were found in the Wyalkatchem and Halberd cultivars (**Figure 2A**), and Wyalkatchem also had the most significant number of differentially expressed lncRNAs in both meiosis and tetrad (**Figures 2A, B**).

Although the majority of lncRNAs displayed cultivar-specific expression patterns, we did identify a subset of lncRNAs that were commonly expressed between heat-sensitive cultivars (245), heat-tolerant cultivars (81), and all four cultivars (105) during meiosis (**Figure 2A**). Additionally, our analysis showed that while most of the identified lncRNAs were specific to either meiosis or tetrad stages, 1,173 lncRNAs exhibited common patterns of upregulation or downregulation between these two developmental stages (**Figure 2C**).

**Figure 3:**
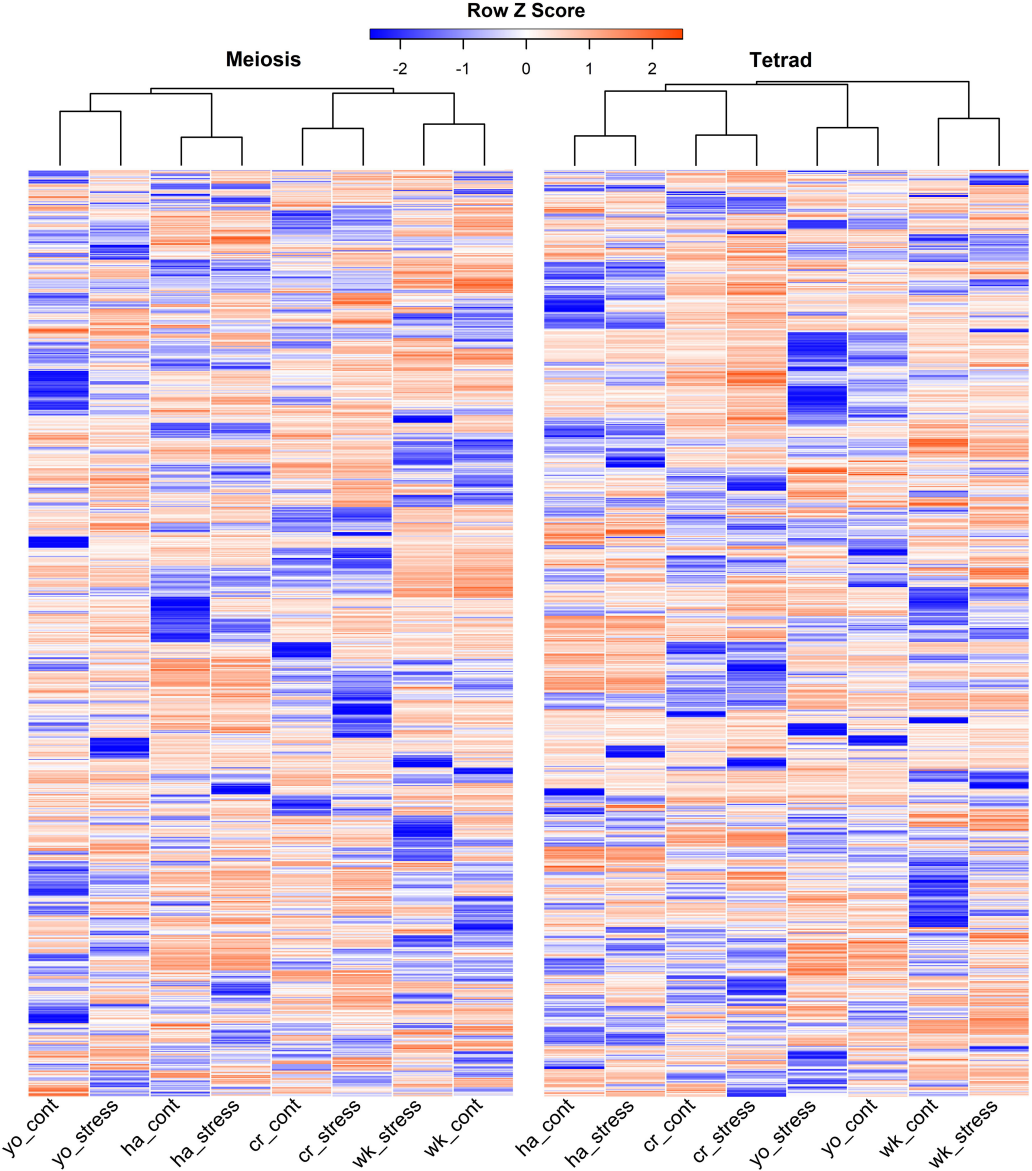
Top 1,000 highly variable expressed lncRNAs during pollen development in response to heat stress in heat-sensitive (cr, Cranbrook and wk, Wyalkatchem) or heat-tolerant (ha, Halberd and yo, Young) wheat cultivars. There was a high level of variability in the expression of lncRNAs, both between stress and control conditions and across different cultivars, during both meiosis and tetrad stages of pollen development.

It’s interesting to note that across all cultivars, we discovered a considerable variation in the expression of lncRNAs during pollen formation under heat stress. To further illustrate this, we present the expression pattern of the top 1,000 lncRNAs with the highest variability during pollen development in **Figure 3**. The heat-shock marker genes found to be upregulated in our analysis were also extracted through the differential expression analysis, and their details are provided in **Supplementary Table S4**. Among those, the most highly upregulated gene encodes chaperone protein and small heat shock protein HSP20/Alpha crystallin. Other notably upregulated genes encode members of the Heat shock factor (HSF) family.

### 3.3 Exploring the role of heat-responsive lncRNAs as *cis*- or *trans*-acting regulatory molecules

**Figure 4:**
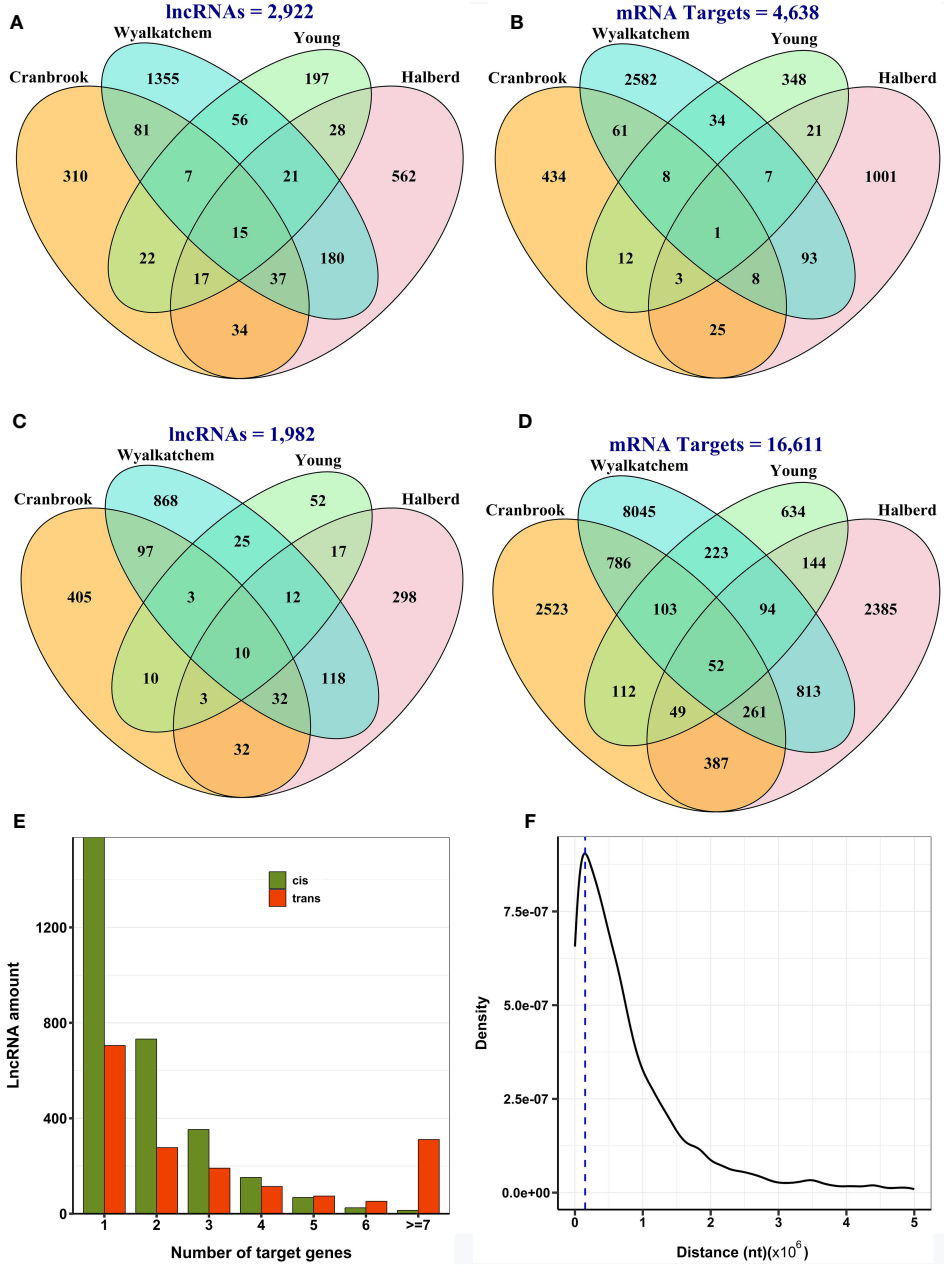
Predicted co-expression of lncRNAs and protein-coding genes in wheat. **(A)** The number of lncRNAs with *cis*-regulatory function. **(B)** The number of target protein-coding genes of lncRNAs in *cis*. **(C)** The number of lncRNAs with *trans*-regulatory function. **(D)** The number of target protein-coding genes of lncRNAs in *trans*. Most lncRNAs and their target genes showed cultivar-specific expression patterns in both *cis* and *trans*-regulatory mechanisms. **(E)** The number of target genes of lncRNAs in *cis* and *trans*, indicating that lncRNAs tend to target usually one to three genes. **(F)** The distance between lncRNAs and their target genes in *cis*, with the dashed line representing the peak density within a distance of 150 kb, indicating that lncRNAs generally regulate their target genes in close proximity.

We focused on their regulation of nearby or distal protein-coding genes to gain insight into the potential functions of differentially expressed lncRNAs. We selected ten protein-coding genes located upstream and downstream of the lncRNAs as *cis* targets and all other protein-coding genes as *trans* targets. Pearson correlation coefficients were then calculated to identify co-expression patterns between lncRNAs and their target genes. The results predicted 5,306 significant correlated expressions between 2,922 lncRNAs and 4,638 neighboring protein-coding genes in *cis* (**Supplementary Table S5**; **Figures 4A, B**) and 41,250 meaningful correlated expressions between 1,982 lncRNAs and 16,611 protein-coding genes in *trans* in all cultivars (**Supplementary Table S6**; **Figures 4C, D**). Again, we observed that most lncRNAs and their *cis* or *trans* targets showed a cultivar-specific association. Most lncRNAs exhibited a narrow range of target genes, with most regulating only one to three protein-coding genes. (**Figure 4E**). The peak density of distance between lncRNAs and their target protein-coding genes in *cis* was observed to be approximately 150 kb from the lncRNA. This suggests that lncRNAs often modulate the expression of genes in their immediate genomic vicinity (**Figure 4F**).

**Figure 5:**
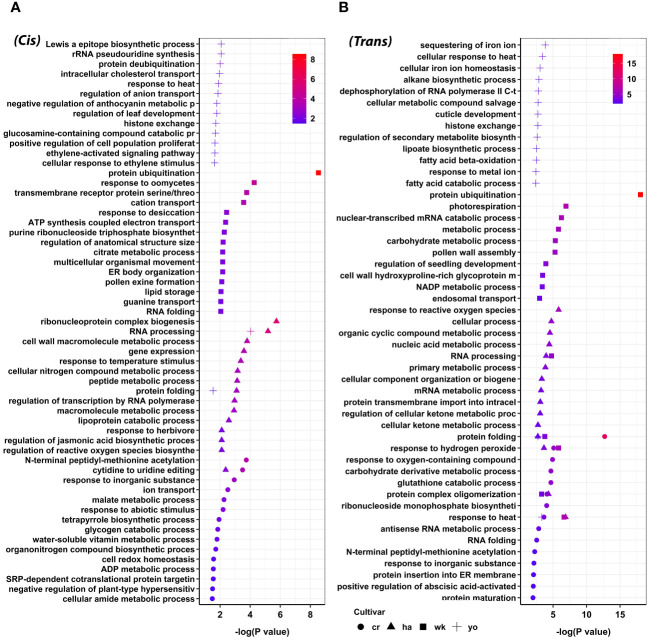
Functional annotation of upregulated target protein-coding genes of lncRNA during pollen development in wheat, categorized based on their *cis* **(A)** and *trans* **(B)** regulatory mechanisms. GO terms such as response to heat, response to hydrogen peroxide, protein complex oligomerization, protein folding, and RNA processing were enriched among different wheat cultivars. Cranbrook (cr) and Wyalkatchem (wk) are heat-sensitive, and Halberd (ha) and Young (yo) are heat-tolerant wheat cultivars.

To investigate the potential regulatory role of lncRNAs in response to heat stress during pollen development in wheat, we conducted GO enrichment analysis on the target protein-coding genes of lncRNAs. Our findings demonstrated the involvement of lncRNAs in a range of biological processes, both as *cis* and *trans*-regulatory elements. For example, we observed that lncRNA target genes exhibiting an upregulation trend in response to heat stress were enriched in 505 GO terms under the biological category of *cis* regulation. These GO terms were further consolidated into 121 parent terms, including RNA processing, response to temperature stimulus, regulation of jasmonic acid biosynthesis, response to abiotic stimulus, response to heat, and protein folding (**Supplementary Table S7**; **Figure 5A**). Enrichment analysis of lncRNA target genes exhibiting a downregulation trend in response to heat stress revealed 674 GO terms under various biological categories. Further consolidation of these GO terms yielded 155 parent terms, encompassing processes such as regulation of cell size, pigment biosynthesis, and chromatin organization (**Supplementary Table S7**).

Similarly, we investigated the *trans*-regulatory mechanism of lncRNAs. We found that the upregulated target genes of lncRNAs were enriched in 941 GO terms. In comparison, the downregulated ones were enriched in 1,019 GO terms, which were subsequently consolidated into 202 and 207 parent terms, respectively (**Supplementary Table S8**). Noteworthy GO terms for the upregulated target genes in *trans* included response to hydrogen peroxide, response to heat, protein ubiquitination, protein folding, and cuticle development (**Figure 5B**). Significant GO terms for the downregulated genes in *trans* were sister chromatid segregation, response to brassinosteroids, lipid modification, and ncRNA catabolic process (**Supplementary Table S8**).

### 3.4 LncRNAs regulate biological processes via lncRNA-miRNA-mRNA regulatory networks

To explore the potential post-transcriptional functions of lncRNAs, we predicted lncRNA-miRNA-mRNA regulatory networks. We identified lncRNA-miRNA modules by predicting interactions between differentially expressed lncRNAs identified in this study and known wheat miRNAs. Subsequently, we identified lncRNA-miRNA-mRNA regulatory networks by analyzing the interactions between selected miRNAs and differentially expressed mRNAs. To identify lncRNAs likely acting as miRNA sponges, we selected those lncRNAs and mRNAs in our lncRNA-miRNA-mRNA networks that showed similar expression trends, such as upregulated lncRNA and upregulated mRNA or downregulated lncRNA and downregulated mRNA. We defined lncRNAs as miRNA precursors if they had perfect complementary sequences with known wheat miRNAs and exhibited opposite expression trends with their target mRNAs, i.e., upregulated lncRNA-downregulated mRNA or downregulated lncRNA-upregulated mRNA. We identified 139 lncRNAs that potentially act as miRNA sponges, regulating the expression of 1,216 protein-coding genes through interactions with 50 miRNAs (**Supplementary Table S9**).

**Figure 6:**
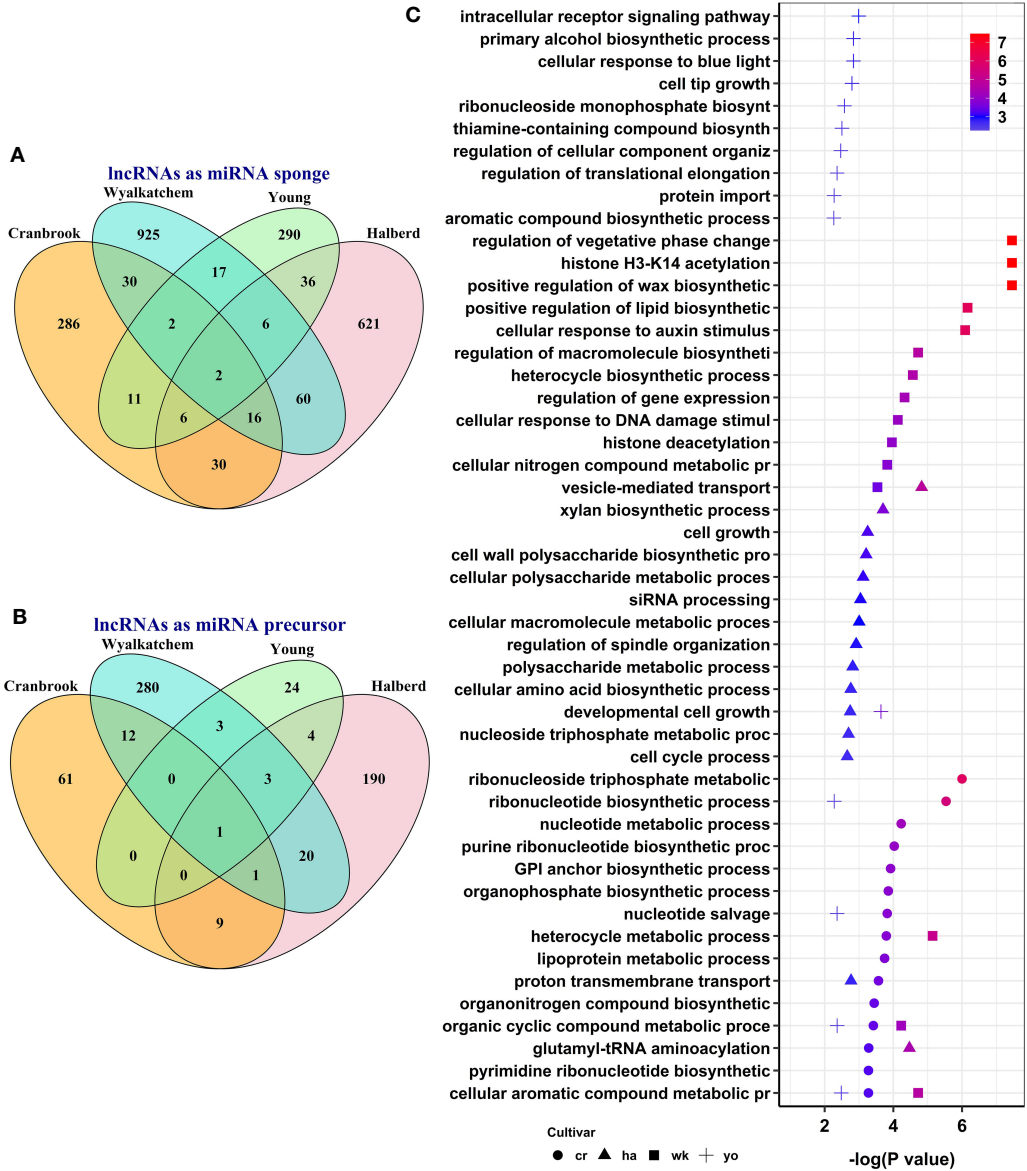
The number and potential function of predicted heat-responsive lncRNA-miRNA-mRNA regulatory networks during pollen development in four wheat cultivars. The number of cultivar-specific and common lncRNA-miRNA-mRNA modules when lncRNAs act as miRNA sponges **(A)** or as miRNA precursors **(B)**. **(C)** illustrates the enriched biological processes for the target genes of lncRNAs in the predicted lncRNA-miRNA-mRNA modules.

Additionally, we identified 25 lncRNAs that could function as miRNA precursors, producing 11 miRNAs that potentially regulate the expression of 438 protein-coding genes (**Supplementary Table S9**). While some lncRNA-miRNA-mRNA modules were shared between two or more cultivars, most were specific to each cultivar (**Figures 6A, B**). Our analysis of miRNA target genes functionally annotated with GO terms revealed that lncRNAs can regulate several biological processes in response to heat stress through post-transcriptional mechanisms during wheat pollen development. Several GO terms were enriched, such as histone H3-K14 acetylation, cellular aromatic compound metabolic process, organic cyclic compound metabolic process, and cellular response to auxin stimulus (**Supplementary Table S10**). **Figure 6C** displays each wheat cultivar’s top 15 enriched GO terms for miRNA target protein-coding genes. While some enriched GO terms were shared across multiple cultivars, others were cultivar specific.

### 3.5 Conservation of lncRNAs varies among plant species based on evolutionary distance

**Figure 7:**
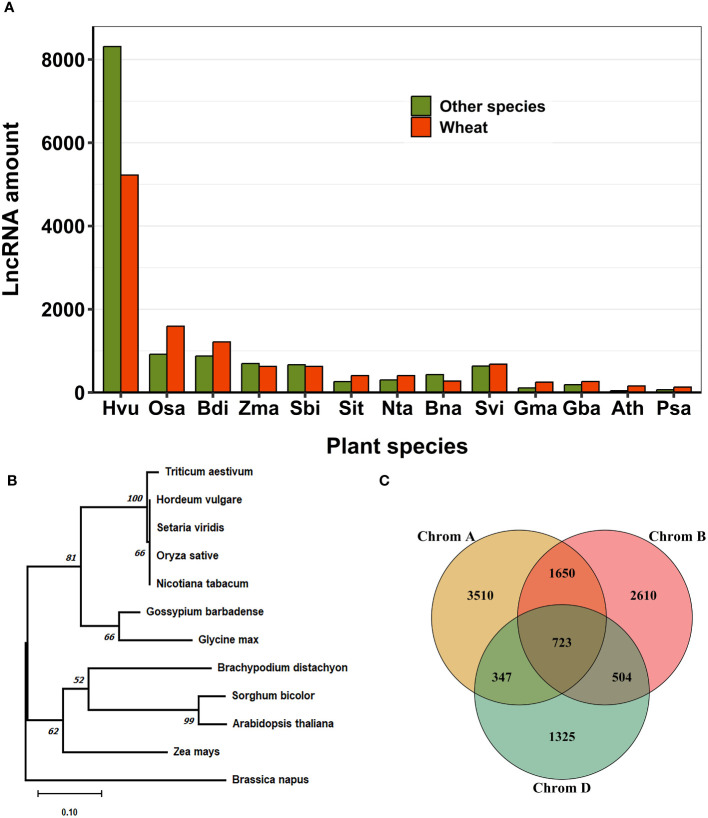
Conservation of lncRNAs in wheat. **(A)** Conservation of lncRNAs was determined by BLAST analysis with 13 other plant species. Most conserved lncRNAs in wheat showed sequence homology with closely related species, *Hordeum vulgare*. **(B)** The Neighbor joining phylogenetic tree with 1000 bootstrap replicates. It represents the evolutionary conservation of a specific lncRNA, MSTRG.56557.1, which is conserved across several plant species. **(C)** The conservation of lncRNAs between A, B, and D wheat genomes. Most lncRNAs exhibit sub-genome-specific expression patterns. Conserved lncRNAs are more common between genome A and B. Initials for plant species in **(A)** *Hordeum vulgare, Oryza sativa, Brachypodium distachyon, Zea mays, Sorghum bicolor, Setaria italica, Nicotiana tabacum, Brassica napus, Setaria viridis, Glycine max, Gossypium barbadense, Arabidopsis thaliana, Pisum sativum.*.

To investigate the evolutionary patterns of lncRNAs, we conducted alignment and conservation analyses between lncRNAs identified in this study and those from 13 plant species available in public databases (**Figure 7A**). Our research revealed that, among the 11,054 lncRNA loci in wheat, 3,263 loci (29.52%) contained conserved sequences with other plant species (**Supplementary Table S11**). Most sequence similarity in lncRNAs was observed between *Triticum aestivum* and *Hordeum vulgare*, followed by *Setaria viridis*, *Brachypodium distachyon*, *Zea mays*, *Oryza sativa*, and *Nicotiana tabacum* (**Figure 7A**). We identified 654 lncRNA loci in wheat that exhibited sequence homology with two or more other plant species. Among these, 20 loci showed homology with more than nine different species. We constructed a phylogenetic tree to investigate the evolutionary conservation of a highly conserved lncRNA, MSTRG.56557.1, identified in wheat and present in 11 other plant species. The phylogenetic tree revealed a close relationship between the identified lncRNA in wheat and those in *Hordeum vulgare*, *Setaria viridis*, *Oryza sativa*, and *Nicotiana tabacum*. (**Figure 7B**). We also investigated the conservation of lncRNAs in wheat’s A, B, and D sub-genomes based on sequence homology (**Supplementary Table S12**). Our study revealed that the majority of lncRNAs exhibit sub-genome-specific expression patterns. However, 3,224 lncRNAs (29.17%) were conserved across all three sub-genomes, with the most frequently expressed lncRNAs shared between the A and B genomes. Additionally, we identified 723 (6.54%) lncRNAs that were conserved in all three genomes (A, B, and D) (**Figure 7C**).

## 4 Discussion

Environmental stresses such as high temperatures limit normal plant growth and development. In response to such stresses, plants activate a series of defense mechanisms that involve changes in the expression of several genes such as HSFA1s, DEHYDRATION-RESPONSIVE ELEMENT BINDING 2A (DREB2A), and JUNGBRUNNEN1 (JUB1) (Zhu, 2016; Ohama et al., 2017). Accumulating evidence reveals that lncRNAs function during normal plant growth and development and play essential roles in response to harsh environmental conditions (Wu et al., 2020b; Urquiaga et al., 2021). Previous studies reported abiotic stress-responsive lncRNAs in several plant species, such as cold-responsive lncRNAs in grapevine (Wang et al., 2019), drought and heat-responsive lncRNAs in *Brassica juncea* (Bhatia et al., 2020), salt stress-responsive lncRNAs in maize (Liu et al., 2022) and rice (Tiwari et al., 2023), and drought-responsive lncRNAs in tomato (Lamin-Samu et al., 2022). In this study, we used transcriptomic data from four wheat cultivars to investigate the dynamic expression of lncRNAs during meiosis and tetrad stages of pollen development in response to heat stress. We identified the expression of lncRNAs from 11,054 loci, of which 5,482 loci showed differential expression patterns. The meiosis stage shows more response to heat stress as the more significant number of expressed lncRNAs and a higher level of changes in differential expression were observed in this stage of pollen development. Our results suggest that stress-responsive lncRNAs are part of the dynamic response of gene expression regulation during pollen development in wheat in response to heat stress (**Table 1**). Meiosis was also noted as the most sensitive stage to heat stress in previous studies (Bokshi et al., 2021). Meiosis processes are more vulnerable to high temperatures, which significantly affect how developing pollen functions as it matures. This sensitivity limits the quantity and quality of mature pollen (Bokshi et al., 2021). In wheat, high temperatures cause changes in pollen development, such as the breakdown of cells during meiosis, leading to two main outcomes. Firstly, the pollen grains might not develop properly, remaining immature. Alternatively, if the initial cell division is successful, there may be difficulties in progressing to the usual three-cell pollen grains (Saini et al., 1984; Bokszczanin et al., 2013). Additionally, heat stress during meiosis results in a decrease in pollen dispersal and noticeable irregularities in pollen shape, groups of pollen grains sticking together, and the formation of pollen grains with multiple nuclei (Jäger et al., 2008; Bokszczanin et al., 2013).

**Table 1:** Selected upregulated lncRNAs in response to heat stress as heat tolerance or heat susceptible biomarkers in wheat.

Cultivar	Dev. Stage	LncRNA locus ID	Log2FC	LncRNA conservation (identity %)
**Halberd/Young**	Meiosis	MSTRG.93120	4.58/2.70	Bdi (75)
		MSTRG.66077	3.17/2.66	–
		MSTRG.4847	3.08/4.52	–
		MSTRG.51292	4.15/2.67	Huv (82)
		MSTRG.16221	3.69/2.72	–
	Tetrad	MSTRG.94397	3.80/3.62	Huv (86)
		MSTRG.53383	2.48/3.64	Huv (82)
		MSTRG.43348	2.52/3.55	–
		MSTRG.80946	1.86/3.37	–
		MSTRG.33763	1.88/3.06	–
**Cranbrook/Wyalkatchem**	Meiosis	MSTRG.6392	2.41/6.85	Huv (98), Svi (97), Osa (97), Nta (97)
		MSTRG.83325	3.09/5.51	–
		MSTRG.52667	4.56/2.83	–
		MSTRG.14617	3.02/3.44	Huv (78)
		MSTRG.10157	3.10/3.14	–
		MSTRG.3277	2.20/4.06	–
	Tetrad	MSTRG.8752	5.97/4.90	–
		MSTRG.105350	5.08/1.55	Huv (83), Zma (75), Svi (74), Sbi (73)
		MSTRG.18574	2.99/2.29	–
		MSTRG.47840	0.92/0.90	–

Symbol "-" : homolog of lncRNA was not found in other plant species.

LncRNAs can function in *cis* to regulate the expression of their target genes in their proximity or in *trans* to regulate distal target gene expression (Rinn and Chang, 2020). However, in lncRNA identification pipelines, functional characterization of a lncRNA is still one of the most challenging tasks. The reason is that there are only a few lncRNAs validated functionally, and the inter-species sequence conservation among lncRNAs is lacking (Golicz et al., 2018b). As lncRNAs usually co-express with their target protein-coding genes, the primary method for lncRNA annotation is to identify the co-expression network between lncRNAs and protein-coding genes, and then, the potential function of lncRNAs is predicted based on the functional analysis of lncRNA’s targets (Xu et al., 2018; Gasparis et al., 2021). With this regard, lncRNAs have been identified as *cis*- or *trans*-acting regulatory molecules in various processes in response to environmental stimuli (Urquiaga et al., 2021). For example, the target genes of *trans*-acting lncRNAs induced under salt stress in duckweed were related to amino acid metabolism, hormone metabolism, cytochrome P450, and CHO metabolism (Fu et al., 2020). In response to drought stress during pollen development in tomatoes, *cis*-acting lncRNAs were found to be involved in abscisic acid (ABA) and jasmonic acid (JA) metabolism, sucrose, and starch metabolism, and tapetum development (Lamin-Samu et al., 2022). In this study, we also investigated the *cis* and *trans* function of lncRNAs in the regulation of gene expression in pollen in response to heat stress. We noted that lncRNAs could modulate gene expression and play roles in various biological processes such as RNA processing, protein folding, protein ubiquitination, regulation of jasmonic acid biosynthesis, and cuticle development (**Table 2** and **Figure 5**). Similar results were also observed in previous studies. For example, up-regulation of genes related to RNA processing and conversion of primary RNAs into mature RNAs were also observed in maize and *Brassica rapa* in response to heat stress (Byeon et al., 2019; He et al., 2019), representing the important function of transcriptional regulation during stress conditions. Protein folding is also vital in living organisms, including plants, as protein function is closely linked to their three-dimensional structures (Miernyk, 1999; Basharov, 2003). Environmental stresses such as heat stress can disrupt the bonds that maintain protein structure, leading to denaturation and loss of function (Freeman et al., 1999; Bischof and He, 2006; Huang and Xu, 2008). Previous studies reported the upregulation of genes related to protein folding in different plant species such as *Brachypodium distachyon* (Chen and Li, 2017), Orchard-grass (Luo et al., 2023) and maize (Wu et al., 2020a). During heat stress, the expression of genes that are related to protein folding and assembly, such as HSPs, can enhance heat tolerance in plants (Miernyk, 1999; Jacob et al., 2017; Ding et al., 2020).

**Table 2:** Selected lncRNAs and their potential target protein-coding genes with specific expression in heat-tolerant or heat-sensitive cultivars.

Cultivars	LncRNA/Target gene	Target gene Ortholog	Target gene GO term and/or description	LncRNA conservation (identity %)
***Cis*** Ha, Yo*	MSTRG.102721/TraesCS7D02G520300	ERD7	Senescence/spartin-associated, response to abscisic acid and abiotic stress	Hvu (92)
	MSTRG.103361/TraesCSU02G047300	Oshsp16.9B	Alpha crystallin/Hsp20 domain, 16.9 kDa heat shock protein 2	Hvu (90)
	MSTRG.26499/TraesCS2D02G150600	CPN10	protein folding, response to heat, GroES chaperonin superfamily	Hvu (81)
	MSTRG.86324/TraesCS6D02G273500	OsMADS57	Transcription factor, MADS-box	–
	MSTRG.90361/TraesCS7A02G308900	RGLG4	jasmonic acid mediated signaling pathway, response to wounding, protein K63-linked ubiquitination	–
**Wk, Cr**	MSTRG.98330/TraesCS7D02G057000	AT4G24340	Nucleoside phosphorylase domain, response to water deprivation, secondary metabolite biosynthetic process	–
	MSTRG.95589/TraesCS7B02G259000	MBF1C	Cro/C1-type helix-turn-helix domain, response to abscisic acid, response to heat	Hvu (100), Svi (100), Osa (99), Nta (99), Zma (98)
	MSTRG.718/TraesCS1A02G072200	WIN1	Pyridoxal phosphate-dependent transferase, arginine metabolic process, primary root development	Hvu (81)
	MSTRG.70350/TraesCS5D02G083300	RPM1	Winged helix-like DNA-binding domain superfamily, defense response	–
	MSTRG.17875/TraesCS2A02G405800	BPS1	shoot system development, root development	–
***Trans*** **Ha, Yo**	MSTRG.105323/TraesCS1B02G137100	OsSET30	Histone H3-K9 methyltransferase	Hvu (84), Zma (80), Svi (78), Sbi (76)
	MSTRG.105323/TraesCS3A02G069900	CHR11	SANT/Myb domain, chromatin remodeling, positive regulation of cellular response to heat	
	MSTRG.105323/TraesCS4A02G358000	REM16	B3 domain transcription factor	
	MSTRG.105323/TraesCS5A02G272400	POM1	Glycoside hydrolase, regulation of salicylic acid metabolic process, response to heat	
	MSTRG.45186/TraesCS6A02G276200	RSL1	Myc-type, basic helix-loop-helix (bHLH) domain, regulation of transcription by RNA polymerase II, root hair initiation	–
**Wk, Cr**	MSTRG.104324/TraesCS2D02G091100	AT5G12010	Serine/threonine-protein kinase, response to abscisic acid and jasmonic acid, response to temperature stimulus	Hvu (84), Zma (78)
	MSTRG.104324/TraesCS7B02G146900	XYLT	Glycosyltransferase 61, post-translational protein targeting to membrane, translocation	
	MSTRG.91627/TraesCS4A02G355200	OsFd3	2Fe-2S ferredoxin-type iron-sulfur binding domain, electron transfer activity	–
	MSTRG.81272/TraesCS2A02G088600	PDC2	Thiamine pyrophosphate enzyme, catalytic activity, cellular response to hypoxia	Bdi (99), Hvu (97), Nta (96), Gma (93), Svi (91)
	MSTRG.29264/TraesCS6D02G197200	AT1G05785	Protein transport protein Got1, endoplasmic reticulum to Golgi vesicle-mediated transport	Osa (99), Nta (94)

*Wk and Cr, Wyalkatchem and Cranbrook (heat-sensitive cultivars); Yo and Ha, Young and Halberd (heat-tolerant cultivars).

Symbol "-" : homolog of lncRNA was not found in other plant species.

LncRNAs can also regulate the expression of target protein-coding genes through lncRNA-small RNA-mRNA interactions. In this regulatory mechanism, lncRNAs can be the substrate for small RNA production, or they can act as competent endogenous RNAs (ceRNAs) and sequester miRNAs from their target mRNAs. Several studies reported the regulatory mechanism of plant lncRNAs through interacting with small RNAs during normal growth and development, or in response to environmental stresses (Yu et al., 2019; Zhang et al., 2019). In rice, for example, lncRNA PMS1T regulates male fertility by acting as a substrate for 21-nucleotide-phased small interfering RNAs (phasiRNAs) (Fan et al., 2016). LncRNA INDUCED BY PHOSPHATE STARVATION1 (IPS1) in *Arabidopsis* inhibits phosphate-starvation–induced miRNA, miR-399, from binding its target mRNA to regulate phosphate homeostasis in the plant (Franco-Zorrilla et al., 2007). The regulatory function of lncRNA-miRNA-mRNA interactions has also been reported in wheat. For instance, in response to drought stress, 10 lncRNA-miRNA-mRNA regulatory modules involving novel miRNAs such as miR417 and miRNA340 have been identified in drought-tolerant and drought-sensitive wheat varieties (Li et al., 2022). Similarly, four lncRNAs TalnRNA5, TahlnRNA27, TapmlnRNA19, and TapmlnRNA8, with upregulated expression in response to heat stress or powdery mildew infection, have been identified as precursors for miRNAs miR2004, miR2066, and miR2010. (Xin et al., 2011). By employing BLAST sequence comparison, TapmlnRNA8 was identified to exhibit sequence similarity with three upregulated lncRNAs in this study, namely MSTRG.20144, MSTRG.31273, MSTRG.51285, and TapmlnRNA19 demonstrated sequence similarity with the upregulated lncRNAs MSTRG.24647 and MSTRG.38376. We also investigated the potential regulatory function of lncRNAs as either miRNA inhibitors or miRNA precursors during wheat pollen development in response to heat stress. We identified 139 lncRNAs inhibiting 50 miRNAs from 1,216 target protein-coding genes and 25 lncRNAs as the precursor for 11 miRNAs targeting 438 downstream protein-coding genes. Among the predicted miRNAs, there were some known stress-related miRNAs, such as miR1122, miR156, miR159, miR160, miR167, miR399, miR408, and miR444. In several plant species, these miRNAs regulate how plants respond to environmental challenges, including salt, drought, and nutrient deprivation (Islam et al., 2022a; Islam et al., 2022b; Singh et al., 2022). For instance, miR160 controls hormonal signaling pathways in response to salt stress in wheat (Lu et al., 2011) and rice (Barrera-Figueroa et al., 2012). We also discovered interactions between lncRNAs and other miRNAs, such as miR1117, miR1125, miR1130, and miR1135, previously identified in wheat heat shock-treated samples (Kumar et al., 2015). We observed that some of the lncRNA-miRNA-mRNA modules were present across several wheat cultivars, indicating the significance of these molecules during the development of heat-stressed pollen. Some of the such lncRNA-miRNA-mRNA modules were summarized in **Table 3**. Functional annotation on the miRNA’s target protein-coding genes revealed that lncRNAs could regulate various biological processes related to plant stress response, several biosynthetic and metabolic processes, protein modification, and hormonal responses. Our results suggest that heat-responsive lncRNAs could regulate pollen development in wheat through the ceRNA mechanism or by producing miRNAs in post-transcriptional regulation processes.

**Table 3:** Selected heat-responsive lncRNA-miRNA-mRNA modules regulating pollen development under heat stress in two or more wheat cultivars.

lncRNA/miRNA/ Protein-coding gene	Target gene GO term and/or description	Cultivars				LncRNA conservation (identity %)
		Wk	Cr	Yo	Ha*	
lncRNA as miRNA inhibitor						
MSTRG.99184/miR1130b-3p/ TraesCS6B02G212900	Ribosome-inactivating protein superfamily, defense response	–	–	✓	✓	–
MSTRG.86324/miR444b/TraesCS6D02G273500	Transcription factor, MADS-box, RNA polymerase II transcription	✓	–	✓	✓	–
MSTRG.96918/miR10516/TraesCS3A02G085200	Leucine-rich repeat domain superfamily, defense response, ADP binding	✓	✓	–	✓	Hvu (88)
MSTRG.22283/miR1127b-3p/TraesCS2B02G527100	Transcription factor IBH1-like, bHLH domain, response to brassinosteroid	✓	✓	–	✓	Hvu (91)
MSTRG.22283/miR1127b-3p/TraesCS3B02G284800	Myc-type, basic helix-loop-helix (bHLH) domain, DNA-binding transcription factor activity	–	–	✓	✓	
MSTRG.75810/miR1120b-3p/TraesCS5B02G204100	SprT-like domain-containing protein Spartan, cellular response to DNA damage stimulus	–	–	✓	✓	–
MSTRG.68371/miR10519/TraesCS2A02G547200	ATP synthase, F1 complex, alpha subunit, proton-transporting ATP synthase complex	✓	✓	✓	✓	Huv (79), Sbi (78)
lncRNA as miRNA precursor						
MSTRG.90453/miR1121/TraesCS6D02G273500	Transcription factor, MADS-box, RNA polymerase II transcription	✓	–	–	✓	–
MSTRG.99184/miR1130b-3p/TraesCS2D02G254200	nucleic acid binding; Group II intron splicing, DEAD/DEAH box helicase domain	–	–	✓	✓	–
MSTRG.91287/miR1128/TraesCS7D02G420400	E3 ubiquitin-protein ligase MBR1/2-like, ubiquitin protein ligase activity	✓	–	–	✓	–

*Wk and Cr, Wyalkatchem and Cranbrook (heat-sensitive cultivars); Yo and Ha, Young and Halberd (heat-tolerant cultivars).

Symbol "-" in column "Cultivars": lncRNA/miRNA/ Protein-coding gene module was not identified in the corresponding Cultivar.

Symbol "-" in the last column: homolog of lncRNA was not found in other plant species.

Symbol "✓" in column "Cultivars": lncRNA/miRNA/Protein-coding gene module was identified in the corresponding Cultivar.

In conclusion, our study reveals widespread and differential expression of lncRNAs in wheat pollen in response to heat stress. We identified 5,482 heat-responsive lncRNAs in meiosis and tetrad stages of pollen development in four different wheat cultivars. Our analysis suggested that lncRNAs could regulate the expression of their target protein-coding genes through *cis* or *trans*-regulatory mechanisms or through functioning as miRNA sequesters or miRNA precursors. Functional enrichment analysis on the target protein-coding genes of lncRNAs predicted the involvement of lncRNAs in many biological processes, including stress-responsive processes during pollen development in wheat. LncRNAs could regulate biological processes such as response to stress, protein modification, protein folding, hormonal response, and various metabolic and biosynthetic processes. We also noted some heat-responsive lncRNAs correlated with protein-coding genes in two or more different wheat cultivars that could be used for functional experiments in later studies. The present study reveals another layer of complex gene regulatory mechanisms in wheat pollen in response to stress conditions. It provides molecular resources and information for future experiments in this field of research.

## Data availability statement

The original contributions presented in the study are included in the article/**Supplementary Material**. Further inquiries can be directed to the corresponding author.

## Author contributions

SB: Methodology, Visualization, Writing – original draft. PB: Conceptualization, Funding acquisition, Project administration, Supervision, Writing – review & editing. MS: Conceptualization, Supervision, Writing – review & editing.
